# How Much Does the Horse’s Hoof Expand?

**Published:** 1882-07

**Authors:** 


HOW MUCH DOES THE HORSE'S HOOF EXPAND?—Drs. A. Lungwitz and H. Schaaf have made some very exact experiments in order to answer the above question. They constructed a shoe on purpose for the measurements, and used it in many experiments. Their results can best be shown in the following table. The widening was measured in millimetres (one millimetre equalling about 1-25 inch).

| Kind of Gait. | No. of Experiments. | Fore Hoof. | | Total Widening. | No. of Experiments | Hind Hoof. | | Total Widening. |
|---|---|---|---|---|---|---|---|---|
| | | Outside Widening. | Inside Widening. | | | Outside Widening. | Inside Widening. | |
| At rest. | 24 | .25 | .27 | .57 | 8 | .25 | .40 | .65 |
| Walk. | 25 | .60 | .75 | 1.37 | 8 | .45 | .56 | .96 |
| Trot. | 55 | .92 | 1.51 | 2.48 | 14 | .40 | .76 | 1.25 |
| Gallop | 8 | 1.21 | 2.00 | 3.28 | 4 | .75 | 1.81 | 2 56 |

These figures show not only the exact amount of lateral expansion, but also that the expansion is rather more upon the inner side. They furnish definite data upon which those interested in the physiology and pathology of shoeing can work.

